# Bimolecular Fluorescence Complementation; Lighting-Up Tau-Tau Interaction in Living Cells

**DOI:** 10.1371/journal.pone.0081682

**Published:** 2013-12-02

**Authors:** HyeJin Tak, Md. Mamunul Haque, Min Jung Kim, Joo Hyun Lee, Ja-Hyun Baik, YoungSoo Kim, Dong Jin Kim, Regis Grailhe, Yun Kyung Kim

**Affiliations:** 1 Korea Institute of Science and Technology (KIST), Brain Science Institute, Center for neuro-medicine, Seoul, South Korea; 2 School of Life Sciences and Biotechnology, Korea University, Seoul, South Korea; 3 Biological Chemistry, University of Science and Technology (UST), Daejon, South Korea; 4 Institut Pasteur Korea, Neurodegenerative Disorders, Sungnam, South Korea; University of South Florida Alzheimer's Institute, United States of America

## Abstract

Abnormal tau aggregation is a pathological hallmark of many neurodegenerative disorders and it is becoming apparent that soluble tau aggregates play a key role in neurodegeneration and memory impairment. Despite this pathological importance, there is currently no single method that allows monitoring soluble tau species in living cells. In this regard, we developed a cell-based sensor that visualizes tau self-assembly. By introducing bimolecular fluorescence complementation (BiFC) technique to tau, we were able to achieve spatial and temporal resolution of tau-tau interactions in a range of states, from soluble dimers to large aggregates. Under basal conditions, tau-BiFC cells exhibited little fluorescence intensity, implying that the majority of tau molecules exist as monomers. Upon chemically induced tau hyperphosphorylation, BiFC fluorescence greatly increased, indicating an increased level of tau-tau interactions. As an indicator of tau assembly, our BiFC sensor would be a useful tool for investigating tau pathology.

## Introduction

Abnormal tau aggregation is a primary pathological hallmark in Alzheimer’s disease (AD) and multiple other neurodegenerative disorders, collectively called tauopathies [1]. In a healthy neuron, tau stabilizes microtubules by promoting axonal outgrowth and neuronal cell polarization. When pathologically hyperphosphorylated, tau dissociates from microtubules and aggregated [2]. For many years, evidences have suggested of a structural framework for tau aggregation, from soluble monomers to insoluble filaments, which then associate into higher order structures, called neurofibrillary tangles (NFTs). Though the pathophysiological importance of NFTs in tauopathies, the causes and molecular mechanisms responsible for triggering the process remain largely unknown. Progress has been slow because there is no reliable method for monitoring tau aggregation in physiological conditions. Most of the studies on tau aggregation have been conducted in non-physiological conditions by using purified tau or tau fragments. Moreover, due to its extreme solubility, tau aggregation needs to be induced artificially by adding cofactors such as heparin. A cell-based model that could monitor tau assembly in living cells would be a useful tool to investigate tau pathology and to discover methods to prevent and reverse the process. 

Full-length human tau contains a microtubule-binding domain consisting of four conserved sequence repeats. Positively charged residues in the sequence repeats are important for binding with the highly negatively charged microtubules (20-30 electrons per αβ-tubulin dimer) [3,4]. Tau’s binding affinity for microtubules is also actively controlled by phosphorylation, which drives dynamic rearrangement of the microtubule network. Abnormal tau hyperphosphorylation disrupts the balance and dramatically reduces its affinity for microtubules [5,6]. Pathogenically, abnormally hyperphosphorylated tau and the aggregates are found in AD brains. As such, hyperphosphorylation is generally considered the cause of tau aggregation. However, this relationship has not yet been fully demonstrated due to the extreme solubility of hyperphosphorylated tau. Regardless of spontaneous or induced hyperphosphorylation, over-expressed tau shows little intrinsic tendency to aggregate in most cell lines [7-9]. To investigate the missing link between tau phosphorylation and aggregation, we focused on the soluble tau aggregates. Recent studies have suggested that tau oligomers induce memory impairment and neuronal degeneration [10,11], and is becoming widely accepted that soluble species of tau might actually be toxic to neuronal cells. 

To visualize tau-tau interactions, we have established a tau-BiFC cell model. The BiFC is a method to visualize protein-protein interactions that is based on the formation of a fluorescence protein complex from non-fluorescent constituents attached to proteins of interests [12]. Previously, a split green fluorescent protein (GFP) complementation technique was used to quantify tau aggregation [13,14]. In the assay, tau is fused to a smaller GFP fragment (GFP 11), and co-expressed in cells with a larger GFP fragment (GFP 1-10). When tau exists as a monomer or low degree aggregate, the large GFP fragment is able to access the small GFP fragment fused to tau, leading to the association of the fluorescently active GFP. When tau aggregates, the reconstitution of active GFP is prohibited and GFP fluorescence decreases in cells. As a method of quantifying aggregation, the split-GFP assay has been highlighted, however, (the scope and resolution of the assay is limited) the limited scope and resolution of the assay do not allow the monitoring of tau oligomers. To overcome this limitation, we have implemented Venus-based BiFC technique by fusing the non-fluorescent N- and C-terminal compartments of Venus protein to tau. As a fluorescence “turn-on” approach, there is no fluorescence when tau exists as a monomer and Venus fluorescence turns on when tau assembles together. By eliminating the background noise from monomeric tau, we were able achieve spatial and temporal resolution of tau (aggregation) dimerization and oligomerization in living cells without the need of staining with exogenous molecules. 

## Results and Discussion

### Establishment of the tau-BiFC sensor

**Figure 1:**
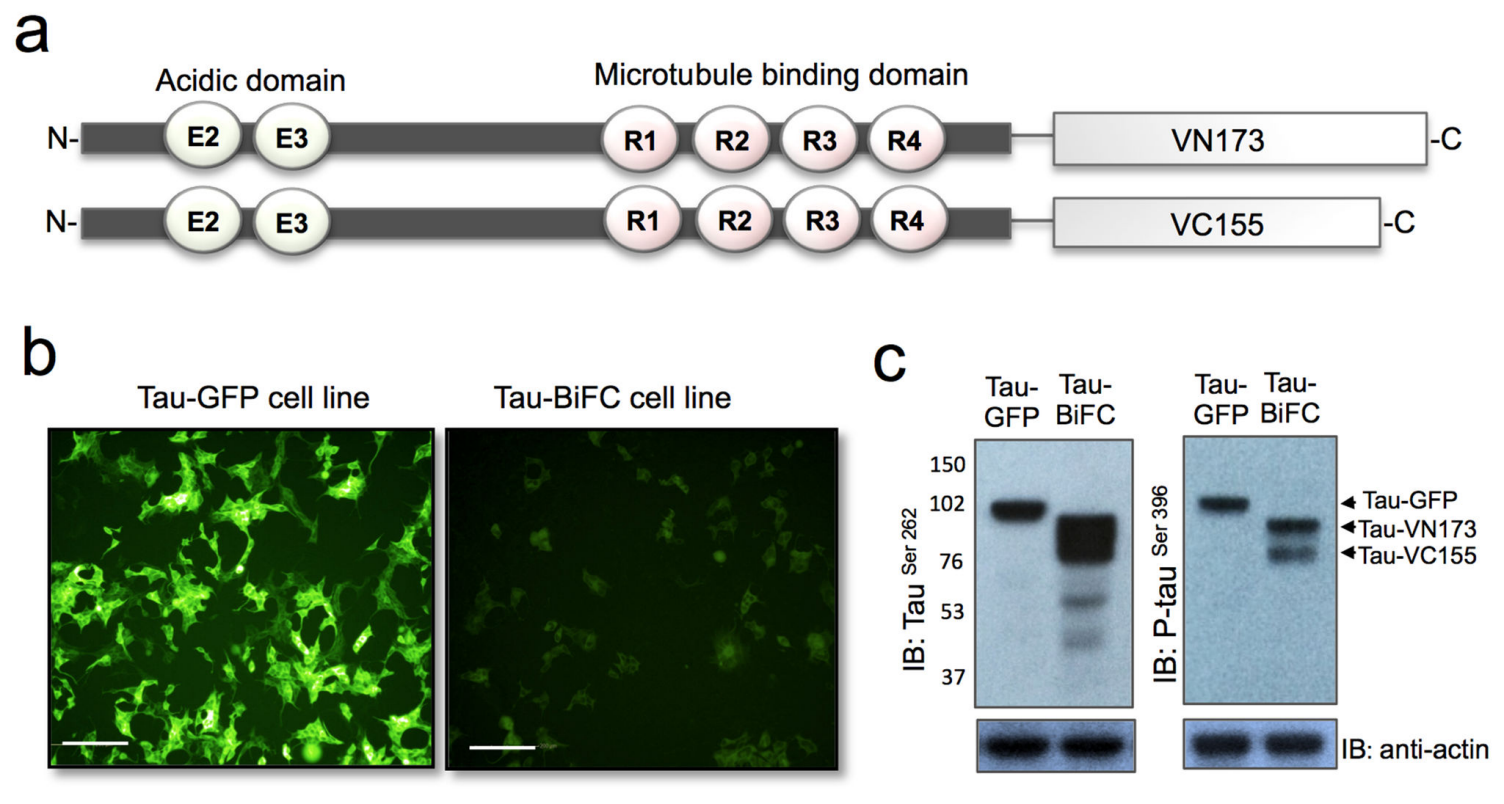
Establishment of HEK293-tau-BiFC cell line. (a) N- and C-terminal constituents of Venus protein was fused to full-length tau (441 a.a.). (b) Basal fluorescence intensity of tau-GFP and tau-BiFC cell line. Scale bar = 200 μm. (c) Expression and basal phosphorylation levels of tau-GFP and tau-BiFC cell line. Immunoblot with anti-tau (ser 262) antibody indicates the expression levels of total tau and immunoblot with anti-tau phospho (ser 396) antibody indicates the basal level of phosphorylated tau. Anti-actin indicates loading controls.

To establish the tau-BiFC sensor, we used a Venus-based BiFC system. The Venus protein is a variant of yellow fluorescence protein (YFP), and is well suited for achieving spatial and temporal resolution of tau assembly because (i) it has fast and efficient maturation, (ii) its self-assembly rate is low compared to that of other BiFC pairs, and (iii) the fluorescence intensity of Venus-based BiFC is 13 times higher than that of EYFP-based BiFC [15,16]. To establish the Venus-based tau-BiFC sensor, full-length human tau (441 a.a.) was fused to the N-terminal fragment of Venus (1-172 a.a., VN173) and the C-terminal fragment of Venus (155-238 a.a., VC155). Two DNA constructs of tau-BiFC were prepared and stably expressed in HEK293 cells (Figure 1a). Then, HEK293-tau-BiFC cells were sorted using fluorescence cytometry to select cells expressing both BiFC constructions (Figure S1 in File S1). A HEK293-tau-GFP cell line was also prepared for comparison. To compare the expression levels of recombinant tau, cell lysates were prepared and subjected to SDS-PAGE analysis. Immunoblot analysis using tau antibodies on cell protein extract indicated two bands for tau-BiFC around 85 and 76 kDa and one band for tau-GFP near 100 kDa (Figure 1c). Importantly, HEK293 does not express endogenous tau that would reduce the efficiency of tau-BiFC maturation. Immuno-blot analysis with phospho-tau antibody (phospho-Ser396) showed basal levels of tau phosphorylation in both cell lines (Figure 1c). Though the similar level of expression, the fluorescence intensity of tau-BiFC cells was considerably low as we envisioned; approximately 19 % of that of tau–GFP (Figure 1b). This implies that the majority of tau molecules expressed in HEK293 exists as monomers at a basal condition. Therefore, tau-conjugated BiFC compartments can not be in proximity to induce BiFC complementation. 

### Tau-BiFC and its association with microtubules

**Figure 2:**
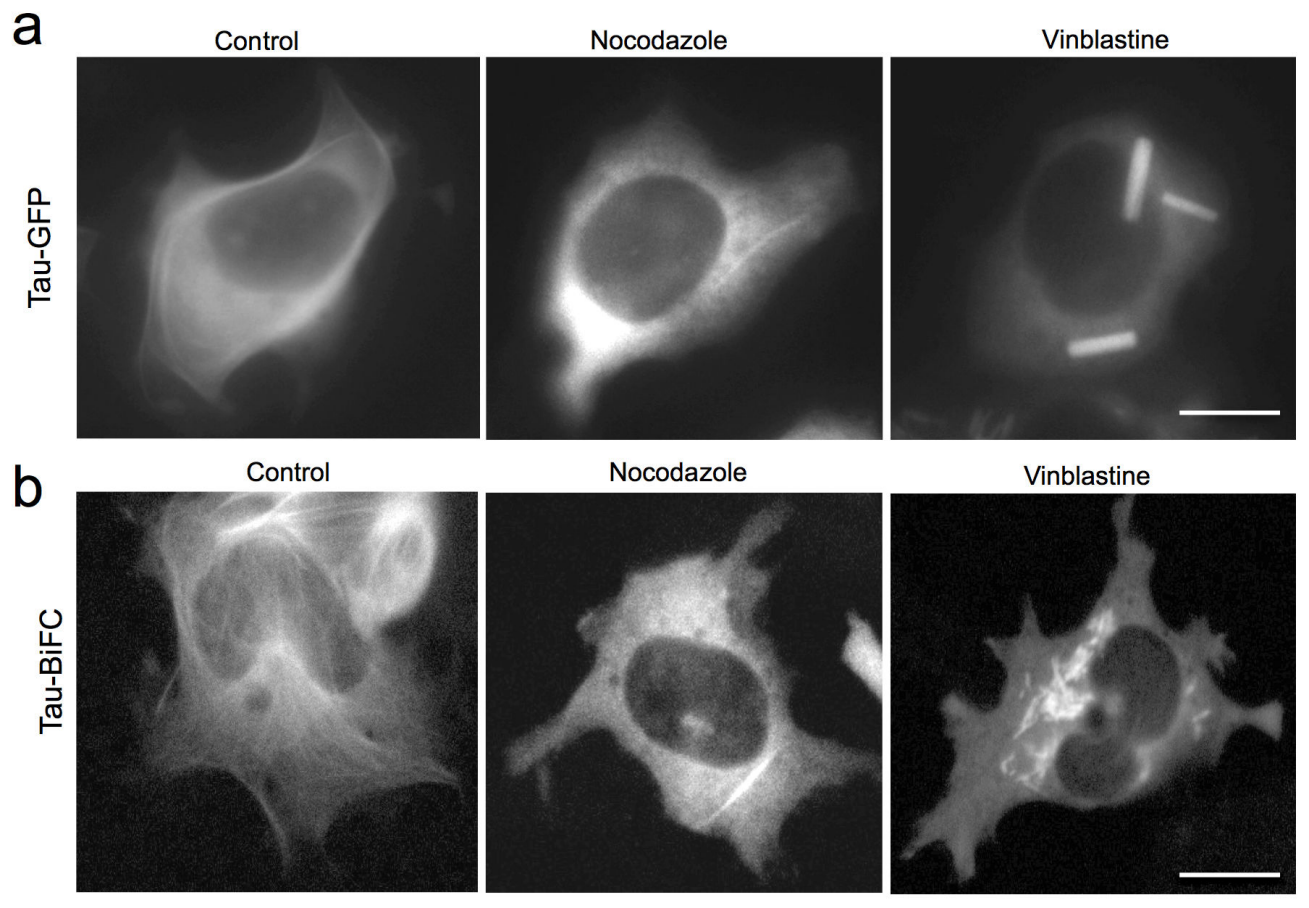
Cellular distributions of HEK293-tau-GFP (a) and HEK293-tau-BiFC (b). Cells were incubated with nocodazole or vinblastine (3 μM) for 30 min and fluorescence images were taken. Scale bar = 10 μm.

Next, we investigated the cellular distribution pattern of tau-BiFC fluorescence. In the case of HEK293-tau-GFP cells, GFP-fluorescence was enriched all over the cytoplasm without showing a strong correlation with microtubules (Figure 2a). In contrast, BiFC-fluorescence, though very faint, showed a clear association with microtubules (Figure 2b). This result implies that highly over-expressed tau binds to microtubules compactly enough to induce BiFC maturation on the microtubules; BiFC maturation occurs only when two compartments are in close vicinity (less than 10 nm) [15,16]. Moreover, strong fluorescence is observed in the cytoplasm of tau-GFP cells but not in tau-BiFC cells. This suggests that the excess tau expressed in the cytoplasm exists as monomers. To further investigate the interaction between tau and microtubules, cells were treated with small molecules that destabilize microtubules. Upon treatment with nocodazole, which dissociates microtubules, microtubule-associated BiFC fluorescence almost disappeared. Upon treatment with vinblastine, which precipitates microtubules, BiFC fluorescence was greatly enhanced on the load-shaped precipitates [17]. Our results clearly indicate that the fusion of BiFC compartments does not interfere with the interaction between tau and microtubules.

### Maturation of tau-BiFC upon tau hyperphosphorylation

**Figure 3:**
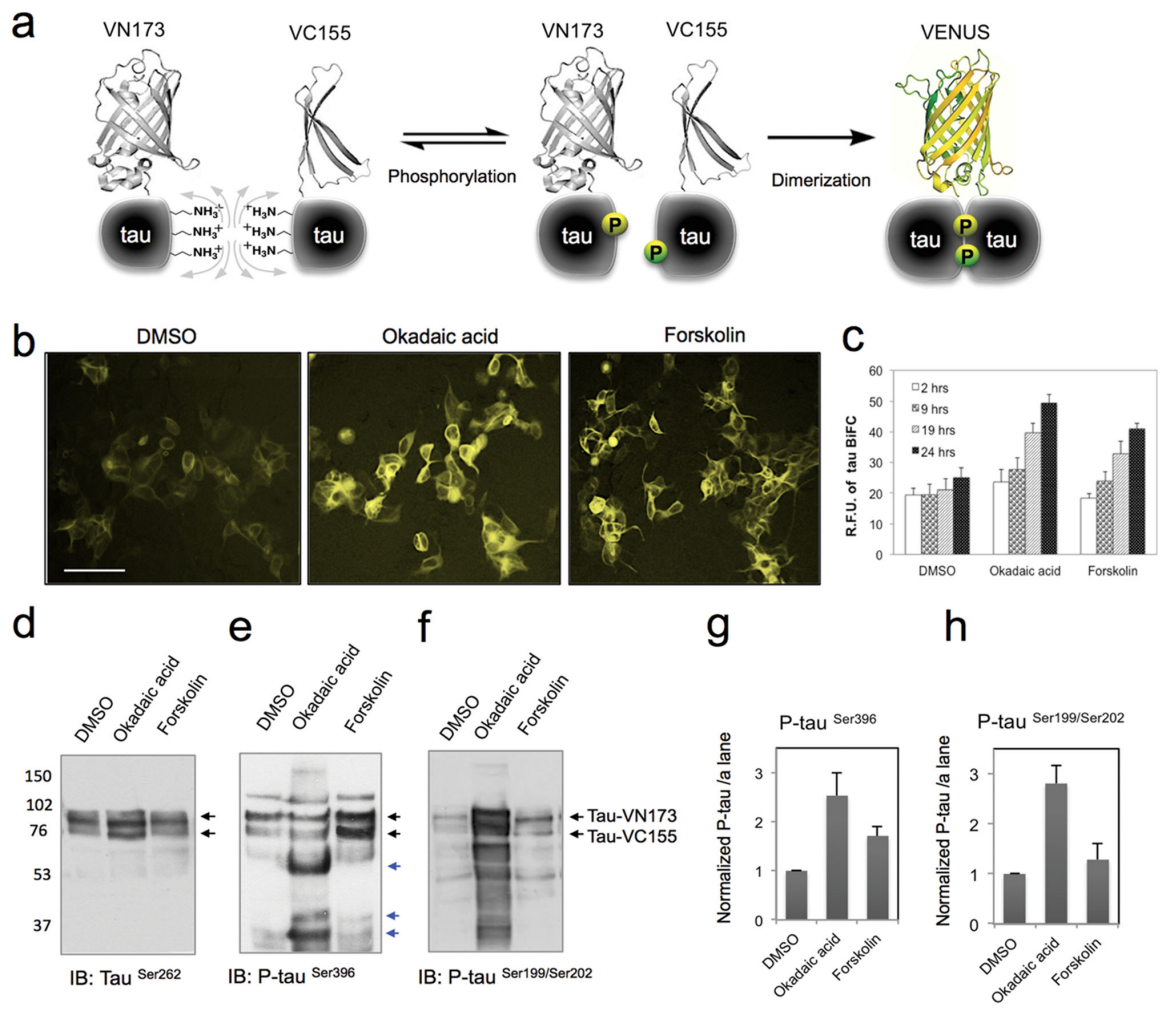
Maturation of tau-BiFC upon tau phosphorylation. (a) Diagram of BiFC maturation upon tau phosphorylation. (b) Tau-BiFC cells were incubated with okadaic acid (30 nM) and forskolin (20 μM) for 24 hrs. Scale bar = 200 μm. (c) Quantification of BiFC-fluorescence increase at various time points. (d-f) For the immunoblot assay, tau-BiFC cells were incubated with compounds for 24 hrs and cell lysates were prepared. Black arrows indicate full-lenth tau, red arrows indicate tau dimers, and blue arrows indicate tau fragments. (g-h) The relative amount of phosphorylated tau including its cleaved forms was normalized with that of non-phosphorylated tau (TauSer262). Error bars indicate s.d. from two independent experiments.

To validate the feasibility of tau-BiFC as an indicator of tau assembly, tau-BiFC cells were treated with forskolin and okadaic acid, which are known to induce tau hyperphosphorylation. Full-length tau has 79 putative serine and threonine residues, and 5 tyrosine residues. Phosphorylation of these residues is tightly regulated by protein kinases and phosphatases to maintain the microtubule dynamics required for neuronal plasticity. Among those regulating enzymes, the major tau phosphatase of the human brain is protein phosphatase 2A (PP2A), which regulates the activities of several protein kinases that phosphorylate tau. Okadaic acid is a potent inhibitor of PP2A, and is known to induce Alzheimer-like tau phosphorylation in rat brains [18,19]. Protein kinase A (PKA) activation by forskolin is also known to induce tau hyperphosphorylation and memory impairment in rat brains [20,21]. As we envisioned, BiFC fluorescence was greatly increased when tau-BiFC cells were incubated for 24 hours with okadaic acid and forskolin by 2.2-fold and 1.9-fold respectively (Figure 3b, c). The rise of BiFC fluorescence directly indicates increased levels of tau-tau interaction in the cells.

**Figure 4:**
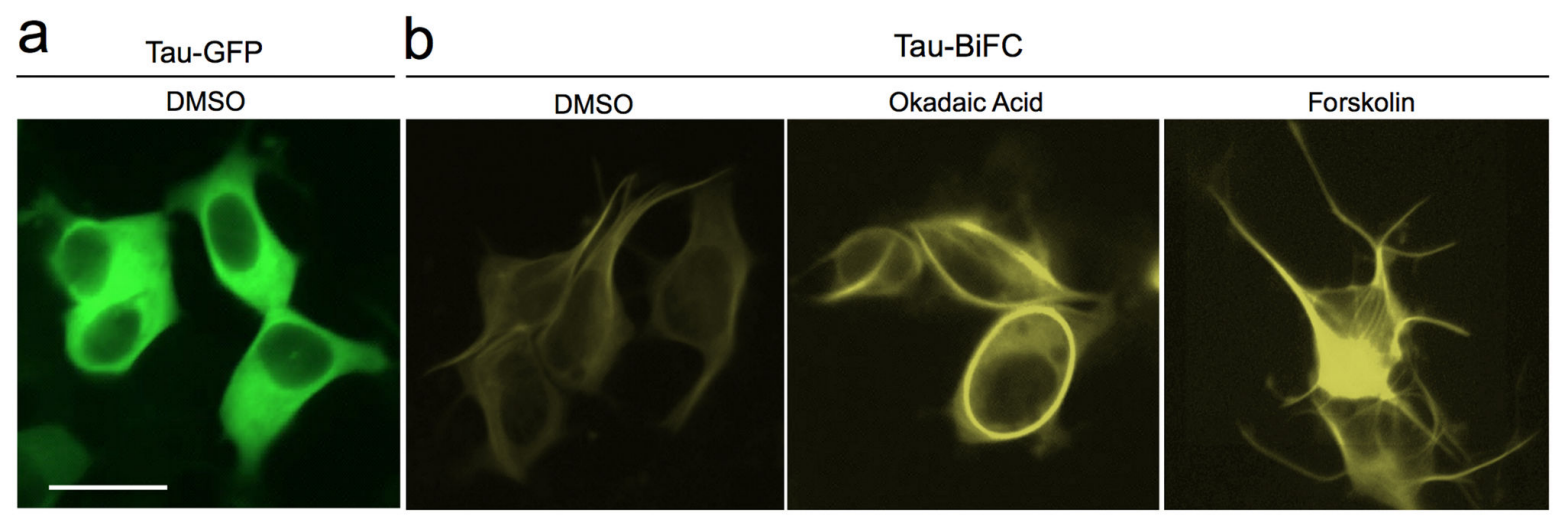
Cellular distribution of tau-BiFC fluorescence. (a) HEK293-tau-GFP control (b) HEK293-tau-BiFC cells were incubated with each compound for 24 hrs.

An immunoblot assay showed that upon okadaic acid (30 nM) treatment, phosphorylation level increase at Ser199 and Ser202 of the full-length tau and a number of tau fragments (Figure 3f), suggesting that okadaic acid induces hyper-phosphorylation of tau. Also, some tau fragments showed strong phosphorylation at Ser396 (indicated by blue arrows in Figure 3e) while full-length tau did not (Figure 3e), implying that cleaved fragments of tau become susceptible substrates for other protein kinases. More importantly, a possible band of tau dimer that was phosphorylated on Ser199, Ser202, and Ser396 was observed around 150 kD (indicated by a red arrow in Figure 3e, f). These results suggest that okadaic acid treatment initiates tau pathology characterized by abnormal tau hyperphosphorylation, and fragmentation, and dimerization. In addition, thread-like tau-aggregates were observed in okadaic acid-treated cells (Figure 4, Figure S2 in File S1), and immuno-fluorescence staining showed that the aggregates were phosphorylated at Ser199 and Ser202 (Figure S3 in File S1). These results support that okadaic acid treatment induces tau pathogenesis in tau-BiFC cell model.

In contrast, while forskolin (20 μM) treatment also induced tau phosphorylation at Ser396 (1.7 fold), but neither tau fragmentation nor dimerization was not observed on the immunoblot (Figure 3e). Although forskolin induced tau phosphorylation and assembly, these effects might not be enough to facilitate tau pathogenesis as shown in okadaic acid treatment. As an activator of adenylyl cyclase, forskolin stimulates cyclic AMP (cAMP) dependent signalling cascades, which have been implicated in a wide range of cellular processes, including transcription, metabolism, cell cycle progression and apoptosis. Especially in neuronal cells, it is well known that forskolin-induced cAMP activation promotes neuronal differentiation and neurite outgrowth [22-24]. Even in the human embryonic kidney (HEK) 293 cells expressing tau-BiFC constructs, forskolin treatment prominently increased neurite-like structures (Figure 4a). Forskolin and okadaic acid are small molecules commonly used to induce tau phosphorylation, however, their contribution to tau aggregation has not yet been clearly identified. By visualizing tau-tau interactions, we could monitor and quantify the effects of small molecules on tau assembly directly in living cells.

## Conclusions

Since Alois Alzheimer discovered the presence of abnormal fibrous inclusions within neurons in a patient’s brain, neurofibrillary tangles are thought to be the pathological structure of tau. However, recent evidences indicate that (toxic tau aggregates are) smaller, soluble tau oligomers are the toxic aggregates, closely linked with neurodegeneration and memory impairment. The large inclusions might form as a survival strategy to protect neuronal cells by sequestrating the toxic aggregates. As such, preventing tau aggregation becomes a potential strategy to cure neurodegenerative disorders associated with tau. To identify the cause and molecular mechanism of tau aggregation and to reverse the processes, a reliable system capable of monitoring tau self-assembly processes is necessary. Our tau-BiFC system provides a chance to monitor and quantify tau aggregation processes by allowing direct visualization of tau-tau interactions in living cells. So, this tau-BiFC sensor would be a useful tool to investigate tau pathogenesis and to discover methods for the prevention and reversal of the aggregation process.

## Methods

### DNA vector construction

A mammalian expression vector for pCMV6-hTau40-GFP was purchased from OriGene Technologies Inc. (Rockville, MD). To replace GFP with BiFC compartments, pBiFC-VN173 and pBiFC-VC155 were obtained from Addgene (Cambridge, MA) and amplified by using PCR primers containing XhoI/PmeI restriction sequences : (VC155-F) 5’-AATTCGGTCG ACCGAGATCT CTCGAGGTAC-3’, (VC155-R) 5’-CTAGTTGTGG TTTGTTTAAA CTCATCAATG TATC-3, (VN173-F) 5’-ATGACGACAA G CTCGAGGCC GCGAATTCAT CG-3’, (VN173-R) 5’-CTAGTTGTGG TTTGTTTAAA CTCATCAATG TATC-3’. pCMV6-hTau40-GFP and PCR amplified inserts were digested with XhoI/PmeI and ligated to generate pCMV6-hTau40-VN173 and pCMV6-hTau-VC155.

### Transfection and cell line establishment

HEK293 was purchased from ATCC (Manassas, VA) and was grown in Dulbecco’s modified eagle medium containing 10% fetal bovine serum and 10,000 units/mL penicillin and 10,000 μg/mL streptomycin at 37 °C in a humidified atmosphere containing 5% CO_2_. The day before transfection, HEK-293 cells were plated on 12-well plates with OPTI-MEM medium (Invitrogen). In order to generate HEK293-tau-GFP cell line, the cells were transfected with pCMV6-hTau40-GFP by using Lipofectamine®2000 reagent (Invitrogen) according to the manufacturer’s instructions. For the generation of HEK293-tau-BiFC cell line, the cells were co-transfected with pCMV6-hTau40-VN173 and pCMV6-hTau40-VC155. To establish the stable cell lines, the transfected cells were incubated with growth medium containing Geneticin for selection. Then, to enrich population, fluorescent cells were sorted by using FACSAria (BD Bioscience).

### Live cell imaging and analysis

For microscopic image analysis, cells were plated in a black transparent 96-well plate. The next day, tau-BiFC cells were treated with the okadaic acid or forskolin at various concentrations. After, 2, 9, 19, and 24 hrs of incubation, the entire 96-well plate was automatically imaged under same exposure by using Operetta® High Contents Screening System (equipped with a 10X and 20X dry lenses). The cellular intensities of tau-BiFC fluorescence were analysed using Harmony 3.1 software. Error bars indicate s.d. from two independent experiments. Each experiment was performed as triplicate.

### Immuno-blot analysis

HEK293-tau-BiFC cells were incubated with okadaic acid or forskolin for 24 hrs at 37 °C. Then, cell lysates were prepared by using CelLytic M (Sigma) containing protease and phosphatase inhibitor cocktail. 10 μg of the protein lysates were separated on an SDS-PAGE gel (10%) and transferred to PVDF membrane for immuno-blot analysis. All tau antibodies were purchased from abcam (Cambridge, MA): pSer199 and pSer202 (ab4864), pSer396 (ab64193) and Ser262 (ab32057).

## Supporting Information

File S1 **Supporting figures**. Figure S1, HEK293-tau-BiFC cell sorting by FACS. Figure S2, The association and reconstitution of tau-BiFC upon okadaic acid treatment. Figure S3, Colocalization of tau-BiFC fluorescence with anti-phosphorylated tau stain.
(DOCX)
