# Supplementary material for: Bimolecular Fluorescence Complementation; Lighting-Up Tau-Tau Interaction in Living Cells
Source: PLoS One. 2013 Dec 2;8(12):e81682. doi: 10.1371/journal.pone.0081682 (PMC3847076; doi:10.1371/journal.pone.0081682)
Supplement: File S1 — Supporting figures. Figure S1, HEK293-tau-BiFC cell sorting by FACS. Figure S2, The association and reconstitution of tau-BiFC upon okadaic acid treatment. Figure S3, Colocalization of tau-BiFC fluorescence with anti-phosphorylated tau stain. (DOCX) [file pone.0081682.s001.docx]

Supporting Information

Bimolecular fluorescence complementation; Lighting-up tau-tau interaction in living cells

Hyejin Tak^a,b,†^, Md. Mamunul Haque^a,c,†^, Minjung Kim^a^, Joo Hyun Lee ^d^, Ja-Hyun Baik^b^, Young Soo Kim^a^, Dong Jin Kim^a^, Regis Grailhe^d*^, and Yun Kyung Kim^a*^

**Contents**

Figure S1. HEK293-tau-BiFC cell sorting by FACS

Figure S2. The association and reconstitution of tau-BiFC upon okadaic acid treatment

Figure S3. Colocalization of tau-BiFC fluorescence with anti-phosphorylated tau stain


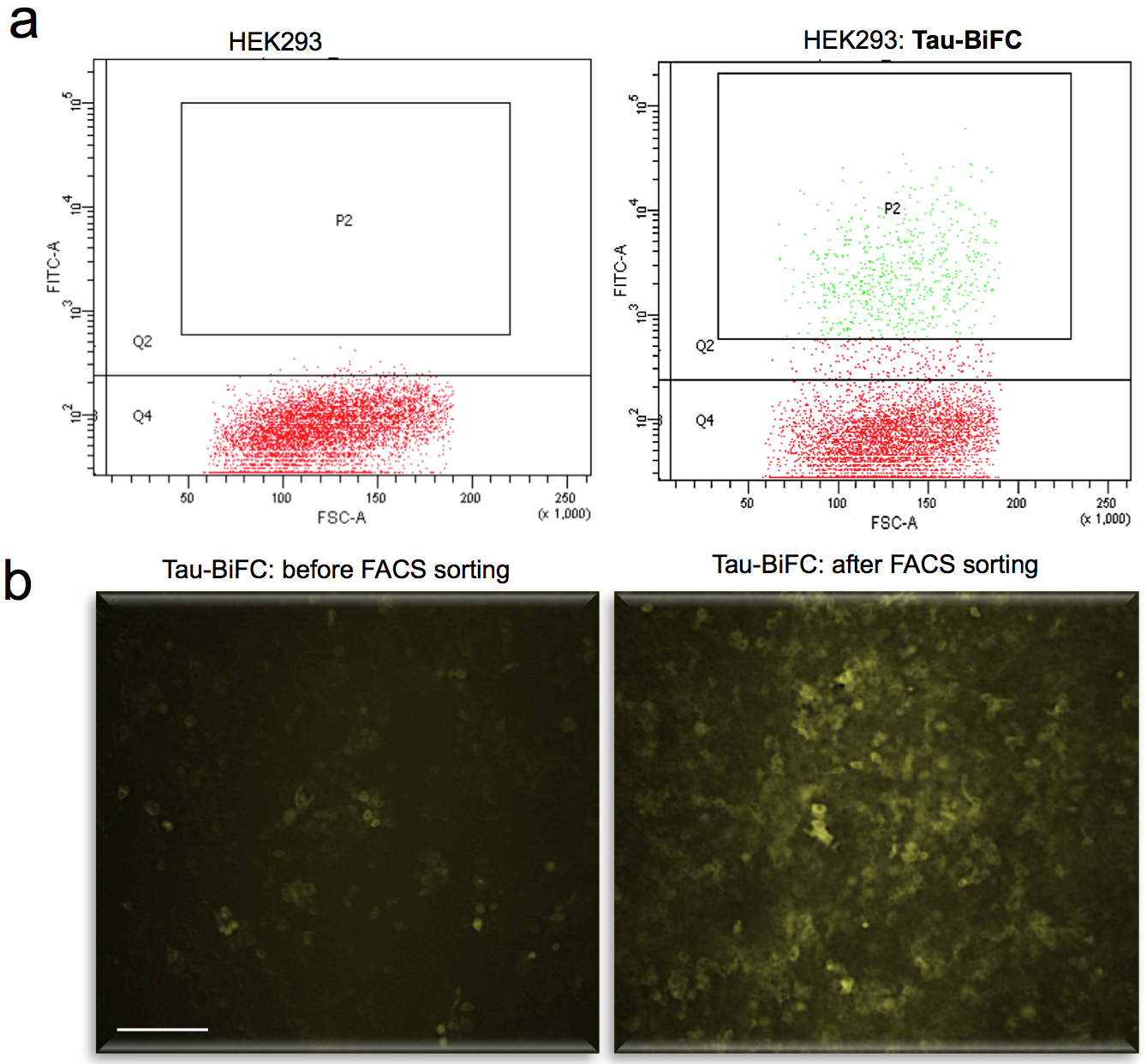


**Figure S1**. **HEK293-tau-BiFC cell sorting by FACS.**

(a) Flow cytometry analysis of HEK293 cells and HEK293 cells expressing tau-BiFC constructs. Transfected cells were primarily selected by using Geneticin and cells exhibiting BiFC-fluorescence were further sorted by using FACS (green dots in P2 region). (b) Fluorescence images of tau-BiFC cells before and after FACS sorting. Tau-BiFC cells showing basal BiFC-fluorescence were greatly enriched after sorting. Scale bar = 100 μm.


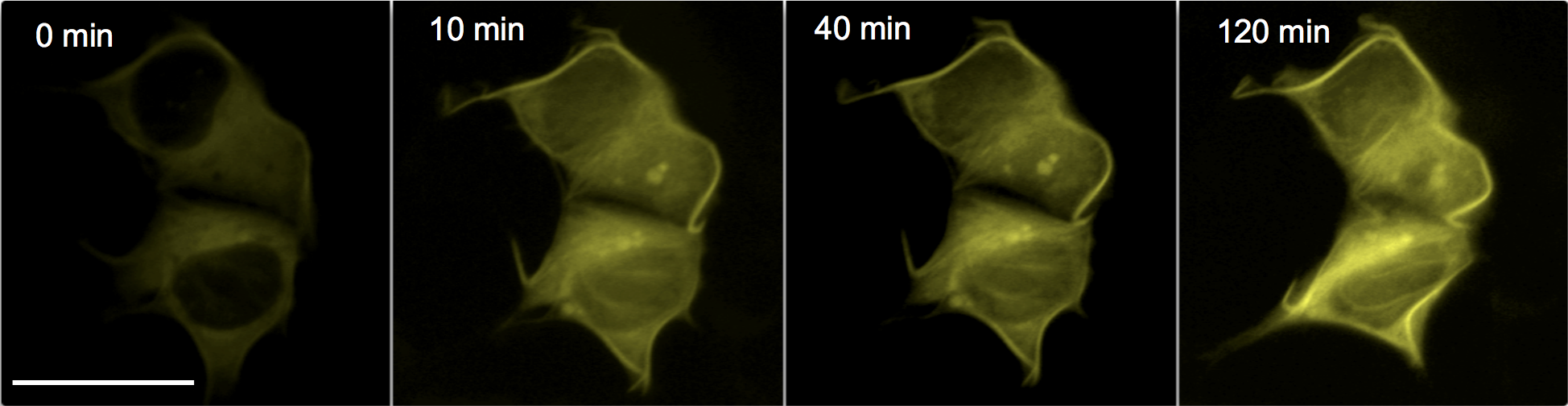


**Figure S2**. **The association and reconstitution of tau-BiFC upon okadaic acid treatment.**

Tau-BiFC cells were incubated with okadaic acid (30 nM) and then imaged by using Nikon microscope. Scale bar = 20 μm.

**
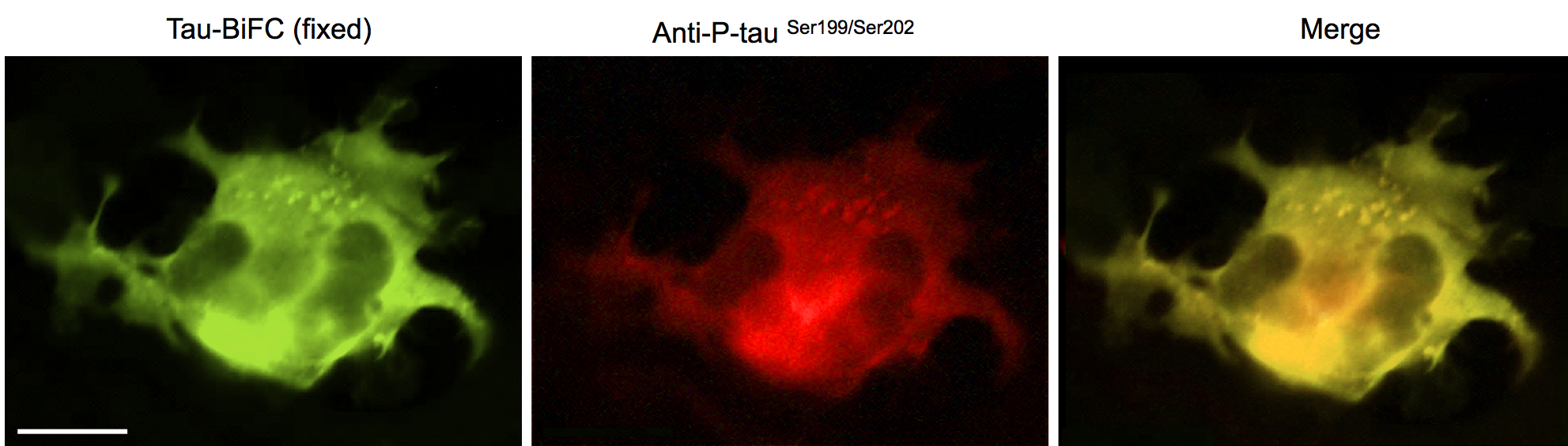
**

**Figure S3. Colocalization of tau-BiFC fluorescence with anti-phosphorylated tau stain.**

Tau-BiFC cells were incubated with okadaic acid (30 nM) for 10 hrs. Then, cells were fixed with 3.7 % paraformaldehyde for immuno-fluorescence stain with anti-phospho tau antibody (Ser199/Ser202). Scale bar = 10 μm.
